# Calculable People? Standardising Assessment Guidelines for Alzheimer’s Disease in 1980s Britain

**DOI:** 10.1017/mdh.2017.56

**Published:** 2017-10

**Authors:** Duncan Wilson

**Affiliations:** Centre for the History of Science, Technology and Medicine, University of Manchester, Room 2.21, Simon Building, Brunswick Street, Manchester M13 9PL, UK

**Keywords:** Medical Research Council, Alzheimer’s disease, Standardisation, Education, Social class

## Abstract

This article shows how funding research on Alzheimer’s disease became a priority for the British Medical Research Council (MRC) in the late 1970s and 1980s, thanks to work that isolated new pathological and biochemical markers and showed that the disease affected a significant proportion of the elderly population. In contrast to histories that focus on the emergence of new and competing theories of disease causation in this period, I argue that concerns over the use of different assessment methods ensured the MRC’s immediate priority was standardising the ways in which researchers identified and recorded symptoms of Alzheimer’s disease in potential research subjects. I detail how the rationale behind the development of standard assessment guidelines was less about arriving at a firm diagnosis and more about facilitating research by generating data that could be easily compared across the disciplines and sites that constitute modern biomedicine. Drawing on criticism of specific tests in the MRC’s guidelines, which some psychiatrists argued were ‘middle class biased’, I also show that debates over standardisation did not simply reflect concerns specific to the fields or areas of research that the MRC sought to govern. Questions about the validity of standard assessment guidelines for Alzheimer’s disease embodied broader concerns about education and social class, which ensured that distinguishing normal from pathological in old age remained a contested and historically contingent process.

## 1 Introduction

Recent decades have witnessed a striking reappraisal of the nature, prevalence and consequences of Alzheimer’s disease. First documented in 1907, when Alois Alzheimer reported cognitive impairment in a middle-aged woman whose brain was found to contain numerous lesions that were known as senile plaques from 1910 onwards, Alzheimer’s disease was widely considered to be rare and secondary to vascular decay, or arteriosclerosis, as a cause of dementia for much of the twentieth century.^1^ Following the discovery of senile plaques in the post-mortem brains of elderly individuals who showed no signs of dementia during life, many psychiatrists even questioned whether it should be considered as a specific disease or an exaggerated form of ‘normal’ ageing.^2^ These attitudes changed dramatically in the 1970s and 1980s, however, when Alzheimer’s disease was increasingly portrayed as a ‘quiet epidemic’^3^ that was the major cause of dementia and ‘one of the leading causes of death in Western countries’.^4^ Psychiatrists, biomedical scientists, medical organisations and politicians such as the former British Prime Minister David Cameron now talk regularly of an Alzheimer’s ‘crisis’ comparable to cancer and HIV/AIDS in that it costs millions of lives a year and places huge strain on national budgets for healthcare and social services.^5^

Several historians and social scientists have detailed the linked factors that underpinned this shift, including: the development of electron microscopy, which facilitated work on the protein structure of senile plaques in the 1960s;^6^ research by British psychiatrists that linked the severity of cognitive impairment to the numbers of senile plaques found during post-mortems, which allowed them to reposition Alzheimer’s disease as a discrete clinical entity and the leading cause of dementia in the 1970s;^7^ the finding that brains of patients contained low levels of the neurotransmitter acetylcholine in the late 1970s, which raised hopes for treatments and led governments across the western world to prioritise research into Alzheimer’s disease;^8^ and the establishment of advocacy groups and media coverage of notable patients, such as former United States President Ronald Reagan, which increased awareness of the social, economic and personal costs of an increasingly ageing population.^9^

But the recent history of Alzheimer’s disease still contains significant gaps, especially when it comes to understanding the ‘strengthening of research efforts’ during the late twentieth century.^10^ While Jesse Ballenger notes that making Alzheimer’s disease a viable target for research involved reaching agreement on what made it a ‘distinct disease entity’ – on the clinical symptoms or pathological markers that differentiated it from other psychiatric conditions and normal ageing – these processes are generally overlooked by histories that detail the increase in research projects from the late 1970s onwards.^11^ This stems from a belief that clinicians and researchers had already demarcated the ‘true boundaries’ of Alzheimer’s disease during the 1960s and early 1970s, as a precondition for later research.^12^ Writers who document research in subsequent decades say little, if anything, about how Alzheimer’s disease was identified or measured in patients and research subjects, and concentrate instead on how researchers elucidated fundamental ideas about disease causation involving biochemical deficits, modified protein cascades and hereditary risk factors.^13^

Drawing on interviews, publications and archival records from the Medical Research Council (MRC), which uses public funds to promote medical and biological research in Britain, this article shows that questions about classification were, in fact, the overriding concern for the British researchers and funding bodies who prioritised work on Alzheimer’s disease during the 1980s.^14^ I outline how researchers who applied for funding utilised a range of different assessments to determine whether patients or research subjects had Alzheimer’s disease, leading the MRC to believe it would be difficult to compare the results obtained across projects. While the MRC initially hoped research on Alzheimer’s disease would be underpinned by what Charles Rosenberg calls an ‘agreed upon disease threshold’, they soon worried that the use of different assessment methods threatened any consensus about how it should be classified or understood.^15^

These concerns prompted the MRC to establish an Alzheimer’s Co-ordinating Committee in 1985, whose overriding aim was to ‘create a more unitary approach to research’.^16^ The Committee organised a workshop where participants agreed on standard assessment guidelines that included questions for a friend or relative, and a series of cognitive tests for the individual who was thought to have Alzheimer’s disease. The MRC emphasised these guidelines were not a tool for clinical diagnosis but were designed to standardise methods by which researchers obtained clinical data, in order to facilitate ‘comparison between research studies’.^17^

The aim of standardising data collection appeared to situate the MRC guidelines within the longer history of cognitive tests, which psychologists, neurologists and psychiatrists had viewed as central to ‘validating therapeutic standards and unifying scientific approaches’ during the early and mid-twentieth century.^18^ Historians have shown how practitioners in these fields developed cognitive tests to consolidate their professional authority, establishing jurisdiction over particular subjects and behaviours while generating ‘comprehensive and calculable’ data that could be shared not just with colleagues but also with government departments, schools or the military.^19^ Yet the MRC’s guidelines were not designed to consolidate authority in a single field and do not sit easily in this literature on cognitive tests and ‘disciplinization’.^20^ They emerged instead from efforts to forge agreement over standards for the collective production of evidence *across* the disciplines and sites that constitute modern biomedicine, ensuring researchers all used the same protocol for documenting the clinical symptoms and biological markers that differentiated ‘normal’ from ‘pathological’.^21^ I argue the MRC’s enthusiasm for uniform assessment methods in research on Alzheimer’s disease therefore highlights what Cambrosio and Keating identify as a ‘will to standardise’ in biomedicine, where funding bodies, researchers and commercial firms develop conventions that impose uniform practices and nosological categories across clinics, laboratories and multi-centre trials.^22^

The MRC’s belief that its guidelines would help researchers produce comparable data rested on a long-standing assumption about the ways in which cognitive tests systematised the encounter between investigators and research subjects. Advocates of cognitive tests had long claimed that assessing performance on tasks whose answers were unambiguously right or wrong provided a stable means of accounting for individual difference and sorting patients into diagnostic groups.^23^ They argued this categorisation was especially reliable because each test had been validated to ensure outcomes bore no trace of an individual’s social circumstances or relationship to the investigator. As Kurt Danziger notes, this meant investigations ‘were not taken as conveying information about an individual-in-a-situation but about an individual in isolation whose characteristics existed outside of any social involvement’.^24^ From the 1950s supporters of cognitive tests included psychiatrists who maintained conditions such as Alzheimer’s disease were not influenced by personal or social factors, as supporters of psychodynamic approaches once argued, but could be ‘explained in entirely material ways, as the outcome of biological processes in the brain’.^25^ By the 1980s, when like-minded psychiatrists helped design the MRC guidelines, cognitive tests were thought to elicit responses that emanated ‘entirely from within the individual’ and underpinned the view of mental illnesses as brain diseases that could be identified through controlled assessments.^26^

But not everyone believed cognitive tests reliably functioned as ‘a social vacuum’.^27^ In the final section of this article, I detail how some psychiatrists wanted changes in the MRC’s guidelines because they contained memory tests that were ‘middle class biased’ and might cause researchers to mistakenly believe that patients with a poor educational background had Alzheimer’s disease.^28^ Portraying certain tests as sources of ambiguity rather than clarity, these critics argued that the MRC guidelines overlooked the ways in which social factors shaped people’s conduct in even the most routinised situations. Challenging those who endorsed a ‘unitary approach’ to assessment, they recommended tests should instead be tailored according to an individual’s educational level or social background.

Scrutinising these arguments does more than simply demonstrate ‘the processes through which conventional standards are proposed, criticized, evaluated and reconfigured’.^29^ It shows that debates about what Timmermans and Berg call ‘the politics of standards’ do not simply reflect concerns specific to the fields or areas of research that funding councils seek to govern.^30^ As we shall see, apparently technical questions about the validity of standard tests for Alzheimer’s disease embodied broader social concerns, and distinguishing normal from pathological in old age remained a contested and historically contingent process.

## 2 Cognitive Testing and ‘The Search for Nosological Clarity’: Setting Priorities for Research into Alzheimer’s Disease

During the 1950s and 1960s, a growing number of figures across disciplines argued ‘medicine is being compelled to an increasing extent to direct its attention to problems that bear some relation to the ageing process’.^31^ Psychiatrists such as Aubrey Lewis and Martin Roth believed mental illness posed the greatest challenge, ‘for the chance of developing it increases with age’, and increasing life expectancies in Britain meant that ‘we must regard the mental disorders of the elderly as likely to be responsible within the next thirty years for the bulk of patients admitted to mental hospitals’.^32^ This was certainly the case with dementia, ‘the most grave and disabling form of mental illness in old age’, which psychiatrists claimed was the main ‘cause of chronicity’ among elderly patients who spent the remainder of their lives on long-stay wards.^33^ Psychiatrists who began to focus on mental illness among the elderly believed their major priority centred on finding ways of accurately differentiating dementia from affective conditions such as depression, which they argued could be treated by electroconvulsive therapy. They viewed identifying these ‘remedial’ cases as essential to easing the burden on psychiatric institutions and, more importantly, to establishing old-age psychiatry as an important field by countering the fatalistic view that all mental illness in the elderly was an irreversible and inevitable consequence of ageing.^34^

While some psychiatrists believed dementia could be easily differentiated from treatable conditions following a clinical examination of the patient and consultation with relatives or caregivers, others warned that diagnosis in this instance hinged on ‘subjective verdicts’ and sometimes varied between practitioners.^35^ In a 1953 paper based on work carried out at Graylingwell Psychiatric Hospital in West Sussex, Martin Roth and Barbara Hopkins claimed that using cognitive assessments psychologists had originally designed for intelligence testing provided a reliable and ‘standardised’ means of distinguishing between dementia and affective conditions such as depression and schizophrenia.^36^ In all cases, they argued, dementia patients scored far lower than other groups in a schedule that became known as the ‘Roth–Hopkins’ test, comprising: the Vocabulary sub-test, where patients had to define certain words; the Digit Span sub-test, where they had to repeat numbers in forward and reverse sequence; the Progressive Matrices test, where they had to identify the missing element in a sequence; as well as more general tests concerning orientation for time and place, and questions relating to well-known events, people and dates. Keen to dismiss the psychodynamic approach endorsed by American psychiatrists such as David Rothschild, who believed dementia arose due to personal factors such as an inability to cope with stress in old age, Roth and Hopkins concluded that cognitive tests underpinned reliable modes of diagnosis which proved mental illnesses were distinct biological entities, or ‘natural kinds’, that could be grouped according to their specific properties and outcomes.^37^ Their argument resonated with British psychiatrists who were keen to establish jurisdiction over conditions such as dementia and, crucially, with figures in the government’s Department for Health and Social Services (DHSS) who viewed accurate rates of diagnosis as essential to allocating resources both in mental hospitals and, after the 1959 Mental Health Act, in the community.^38^ By the late 1960s, as Roth and David Myers outlined in the *British Journal of Hospital Medicine*, cognitive tests had become a routine component of a diagnostic schedule that also included an interview with the patient to determine awareness ‘of their present circumstances… and of current events’, and a discussion with relatives about the onset and severity of symptoms.^39^

Cognitive tests were also used for research in this period, as part of assessment schedules that helped psychiatrists isolate dementia patients and explore correlations between cognitive impairment and neurological or pathological factors.^40^ Psychiatrists often utilised elements from different psychological tests to make up their own assessment schedules for research, which then became part of the growing number of tests that colleagues in Britain and elsewhere used for clinical diagnosis.^41^ For example, as part of research that explored links between cognitive decline and senile plaque numbers, undertaken in the mid-1960s with the pathologist Bernard Tomlinson and Martin Roth following his move to Newcastle upon Tyne, the psychiatrist Garry Blessed designed a schedule that drew on elements of the Roth–Hopkins test and included questions relating to a patient’s ability to recall personal dates or events.^42^ The new ‘dementia scale’, which soon became used for clinical diagnosis, helped Blessed, Tomlinson and Roth establish that while the brains of seemingly healthy patients contained senile plaques, an individual only developed symptoms of dementia ‘once the degenerative process measured by plaque counts passes a certain threshold’.^43^ This finding led Roth and others to claim that Alzheimer’s disease existed as ‘an accelerated and intensified’ form of processes associated with normal ageing, and that further work was needed to establish why plaques accumulated more readily in the brains of particular individuals.^44^

Demands for more research were lent urgency by Blessed, Tomlinson and Roth’s additional finding that senile plaques occurred far more in post-mortem brain tissue than arteriosclerosis, repositioning Alzheimer’s disease as the most common cause of dementia, and by epidemiological surveys which outlined how dementia occurred at ‘considerably higher’ rates than previously assumed, with eight per cent of elderly people tested in Newcastle upon Tyne displaying symptoms of cognitive decline.^45^ Following renewed warnings that hospitalising this many patients would ‘overwhelm’ the National Health Service, the DHSS claimed its ‘top priority’ was funding work on early diagnosis and alternative models of community-based care.^46^ Members of the MRC’s psychiatry committee, including Martin Roth, agreed that dementia posed ‘serious problems for the delivery of healthcare’ and claimed it now ‘merited urgent attention’.^47^ In their 1975 policy review, the psychiatry committee and the neurosciences board agreed to establish a subcommittee tasked with determining which aspects of dementia research the MRC should prioritise.

This subcommittee was chaired by Alwyn Lishman, who, like the vast majority of British psychiatrists, believed that mental illnesses stemmed from biological changes in the brain, while its five other members were drawn from neurology, psychology and neuropathology.^48^ During their first meeting, the subcommittee decided to organise a two-day conference to ‘consult many persons with expert knowledge or particular experience of working in the field’.^49^ Participants at this conference, which included subcommittee members, civil servants from the DHSS and the government’s chief scientist, heard papers on approaches to research in pathology, nutritional science, biochemistry, psychiatry and genetics, as well as more general ‘nosological problems’.^50^

Lishman submitted a draft of the subcommittee’s report, outlining which aspects of research should be prioritised, to the psychiatry committee in March 1976. Its introduction claimed research on dementia had ‘been neglected to an extraordinary degree’ and warned the current ‘lack of understanding is in contrast to the importance of subject, in terms of the devastating nature of the illness and the enormous size and cost of the problem’.^51^ Here, as throughout, the subcommittee adopted the foreboding language increasingly used to describe dementia: presenting it as ‘one of the biggest problems facing the health and social services today, largely as a result of the increased numbers of persons reaching longevity’.^52^ They argued the ‘urgency of the research challenge’ was self-evident, and that overcoming the ‘profound ignorance’ surrounding dementia was vital to resolving ‘the present crises and shortages in healthcare’.^53^

The report also revealed the professional outlook of its members, and British psychiatry more generally, when it detailed how the subcommittee’s recommendations focussed on problems that were ‘biologically-based’ and could be explored by clinical and laboratory research.^54^ While they acknowledged social factors might influence ‘patterns of referral’, subcommittee members overwhelmingly rejected the psychodynamic belief that social or personal factors had ‘any bearing on primary aetiology’. The subcommittee were also clear that their proposals would not prioritise a specific cause of dementia and would encompass all conditions that were known to cause irreversible cognitive decline, including arteriosclerosis, Alzheimer’s disease, Huntingdon’s chorea, Pick’s disease and ‘rarer varieties’ such as Creutzfeld–Jakob’s disease.^55^

When it came to specific recommendations, the subcommittee claimed the MRC’s first priority, which members described as ‘of fundamental importance’, should involve funding work that established ‘clearer demarcation of the disorders from their surrounding territories – from other disease processes which mimic their clinical picture and from the “natural” processes of senescence – and of firmer distinctions between one member of the group and another’.^56^ They argued the existing battery of cognitive tests only helped identify severe cases of dementia, and was of little use when it came to differentiating moderate or mild instances from other conditions and normal ageing, or distinguishing between specific causes of dementia such as Alzheimer’s disease and arteriosclerosis. The subcommittee argued that ‘most urgency attaches to questions of diagnosis and nosology’ because clarification here was vital if researchers in different fields were to ‘know precisely what are the different disease entities to be tackled’. ‘A firm nosology’, they concluded, was essential in order to ‘pave the way for more intensive pathological, biochemical and other laboratory studies’.^57^

In a discussion of the report with the psychiatry committee, Lishman claimed new approaches in computerised tomography (CT) scanning, which allowed clinicians and researchers to visualise the brains of living patients, offered the greatest ‘scope for improvement’ in identifying dementia.^58^ But he conceded that it would be years before CT scanning was ‘readily available’ and urged the MRC to fund improvements in more traditional ‘psychological measurement techniques’ such as cognitive testing.^59^ The psychiatry committee agreed that using intelligence tests for dementia research was ‘often inappropriate’, since these were designed for and validated on young and middle-aged adults with no or little cognitive impairment.^60^ Patients with symptoms of dementia were often unable to register scores or scored in an insensitive part of the range, and the tests provided little information about the severity of their condition, its progress or its causes. This led the psychiatry committee to endorse the subcommittee’s claim that the ‘highest priority’ should be given to researchers who looked to improve tests that measured cognitive decline in the elderly, ‘with the particular aim of clarifying nosological issues’.^61^

When their final report was published in 1977, the subcommittee argued that ‘it should be well within the capability of present-day psychology to devise improved methods of measuring change over time in dementia patients’.^62^ They urged the MRC to prioritise funding for researchers who sought to identify ‘the relevant cognitive parameters to be used in measurement’ and designed tests which could be undertaken ‘by subjects with limited functional capacity’.^63^ These new assessments, the subcommittee concluded, should replace the variety of tests currently in use and be adopted as ‘agreed standards’ by research teams working in hospitals and local authority care homes, or in the community as part of ongoing ‘cohort studies’.^64^

The subcommittee’s report was largely well received. The DHSS argued it should form the basis of a joint policy between the government and the MRC,^65^ while a review in *Nature* agreed that ‘one of the most urgent needs is a clarification of the various types of dementia and their comparison with the normal ageing process’.^66^ A response in *The Lancet* was less positive, however, and criticised the MRC for taking too long to publish the report. Earlier in 1977, a team led by the biochemist Elaine Perry and the neuropathologist Robert Perry had published a paper where they claimed to find depleted levels of acetylcholine in the brains of patients with Alzheimer’s disease.^67^ This raised the prospect of therapeutic intervention, and *The Lancet*’s reviewer argued ‘scientific endeavour has already overtaken some of the conclusions [in the report] and thrust others aside, thus helping to show the problems that are really worth tackling at present’.^68^ Implying that researchers should spend more time looking to treat Alzheimer’s disease and other causes of dementia, rather than trying to classify them, the reviewer concluded that ‘exciting work on dementia continues in Britain (as elsewhere), but it will not be helped by this pamphlet’.^69^

Despite this criticism, researchers continued to prioritise the development of improved cognitive tests. Lishman, who dismissed the review in *The Lancet* as the product ‘of someone with an axe to grind’,^70^ maintained that ‘effective drug trials would not be feasible until techniques of psychological measurement had improved’, as researchers needed an accurate way of gauging changes in symptoms following treatment.^71^ Researchers who applied to the MRC also viewed the development of new cognitive tests as integral to, not distinct from, work on brain biochemistry and drug development. This was clear in 1978, when Martin Roth, now working at the University of Cambridge, requested funding for a ‘multidisciplinary’ project that aimed to correlate clinical observations made during the life of elderly patients with findings made in post-mortem brain tissue, including the number of plaques and levels of acetylcholine.^72^ Roth outlined how one of the project’s main objectives was ‘to develop improved psychometric measures for the assessment of states of dementia’, which would be administered to roughly two hundred patients on the geriatric ward at Fulbourn Psychiatric Hospital in Cambridgeshire.^73^ He argued the intelligence tests used in his previous work were ‘relatively crude’ and ‘not suitable for studies of the kind envisaged’.^74^ While the fact that dementia patients generally failed to register a score in existing tests meant they were useful in ‘separating elderly patients with different forms of psychiatric disorder into relatively distinct groups’, it also meant they were of little use when it came to enabling more ‘precise differentiation’ amongst dementia patients.^75^ Roth claimed ‘more refined measures’ were therefore needed so researchers could identify mild and moderate cases of dementia, assess changes in a range of intellectual functions over time, determine whether an individual’s symptoms were caused by Alzheimer’s disease, arteriosclerosis or a ‘mixed picture’, and establish firm links between clinical and post-mortem observations.^76^

The aims of this proposal clearly overlapped with the priorities set out in the subcommittee’s report, and the MRC awarded Roth and colleagues funding for a three-year project in January 1978.^77^ They used this money to develop a comprehensive test battery known as the ‘Cambridge Mental Disorders of the Elderly Examination’, or CAMDEX, which comprised several sections designed ‘for the early detection of dementia’: a structured interview with the patient on their health and family history, a detailed cognitive examination, an interview with a relative or friend to obtain ‘independent information’ about the patient, and a physical assessment including a CT scan and blood tests.^78^ The cognitive examination formed the bulk of the CAMDEX and took roughly forty minutes to complete. It included questions on the date and location, on the patient’s ability to recognise photographs of everyday items, such as a shoe or a pair of glasses, to copy diagrams of basic shapes, and to respond to commands such as ‘take this piece of paper in your right hand and fold it in half’.^79^

While the CAMDEX was promoted as a new test, like predecessors such as Blessed’s dementia scale it incorporated significant elements of existing tests. Its sections on orientation, object recognition and motor skills (or ‘praxis’) drew on the Mini-Mental State Examination (MMSE), which the American psychiatrist Marshal Folstein had designed in the early 1970s to assess levels of cognitive impairment in elderly patients and distinguish dementia from conditions such as depression.^80^ The MMSE was a brief test that took roughly ten minutes and required no specialist training to administer. This ensured it was widely used by the mid-1980s, and members of Roth’s ‘Cambridge team’, such as the psychologist Felicia Huppert, believed that incorporating it into the CAMDEX ensured their findings would ‘be comparable with those of others’.^81^ But they also argued the CAMDEX tested a ‘wider range of cognitive functions’ and had ‘a number of advantages’ over the MMSE.^82^ These additional components included tests for remote memory and general knowledge, where patients were asked to recall the dates of wars, name public figures such as the Prime Minister and recognise pictures of famous individuals such as the Queen, and tests where patients were asked to demonstrate abstract thought by explaining the relationship between objects such as such as a table and chair.^83^

Roth had long believed the current practice of selecting different cognitive tests for diagnosis or research was problematic, as it meant there was no ‘standardised administration’ across locations and projects.^84^ In promoting the CAMDEX, Roth and colleagues were clear that it could overcome this problem and function as an ‘agreed standard’ that enabled researchers to generate comparable data across different projects, whether in refining ‘internationally agreed criteria’ for mild, moderate and severe dementia, exploring links between cognitive impairment and biochemical change, or seeking to develop ‘pharmacological and behavioural interventions’.^85^ This was clear in 1985, when Felicia Huppert submitted a proposal to the MRC for research that aimed to ‘undertake detailed analysis of attention and memory’ in the early stages of dementia by combining the CAMDEX with new computer-based tests for response time and pattern recognition.^86^ Referees and the MRC grants committee supported the proposal and agreed the CAMDEX would help Huppert generate reliable data on this ‘important’ issue.^87^ But they were less enthusiastic about another application that sought to investigate the clinical picture of early Alzheimer’s disease by using intelligence tests and a scale that measured ‘performances on activities of daily living’.^88^ One referee argued these tests were inadequate and claimed he would ‘be very sorry if, because of diversion of resources to *this* study, it became impossible for better cognitive performance measures to be used, on equivalently documented samples of patients, by other investigators’.^89^

After considering a third proposal which planned to use another battery of tests to investigate the same problem, the grants committee urged the MRC to establish a specialist committee that functioned ‘as a collective referee’ for all applications in this area of research.^90^ They argued the new committee should co-ordinate research by identifying ‘areas of overlap’ between projects, preventing ‘unnecessary duplication of research effort’, and should also encourage ‘opportunities for collaborative research… perhaps by initiating a multi-centred study’.^91^ The MRC neurosciences board responded to this proposal in early 1985 and established a new Alzheimer’s Co-ordinating Committee, chaired by the neurologist Charles Marsden, a former member of Lishman’s subcommittee, with nine other members drawn from psychology, pathology, psychiatry and geriatric medicine.^92^

The title the neuroscience board chose for this new committee was telling. In the period since Lishman’s subcommittee claimed its proposals covered all instances of ‘senile and presenile dementia’, clinicians and researchers had come to widely regard Alzheimer’s disease as the most common cause of dementia.^93^ Work on biochemical deficits raised the possibility of drug treatments, transforming Alzheimer’s disease into a ‘project for modern biomedical science’,^94^ and it was the focus of most research proposals concerning dementia submitted to the MRC in 1984 and 1985.^95^ At their first meeting Marsden’s co-ordinating committee endorsed this focus on Alzheimer’s disease and claimed it was ‘an important area of research’, although they asked the MRC to send them applications on other causes of dementia ‘for information’.^96^

At this initial meeting the new committee also agreed ‘with the assessment of the grants committee that large scale, long-term and multidisciplinary studies were desirable, and probably essential for producing a clear picture [of Alzheimer’s disease] and discovering its cause’.^97^ But they warned that collaboration was presently difficult, ‘if not impossible’, as ‘there appeared to be no consensus… as to the most appropriate methods of clinical assessment’.^98^ Psychiatrists had argued that the choice of cognitive tests affected diagnostic outcomes since the late 1960s,^99^ and Felicia Huppert cited differing estimates of the prevalence of mild dementia, which varied ‘from 2.6% in Britain to an astonishing 52.7% in Japan’, to promote the CAMDEX as a standard assessment in 1985.^100^ The co-ordinating committee drew on these concerns to warn that reliance on different tests meant researchers, the MRC and the government could not reliably compare findings across projects. A decade after the MRC first claimed dementia merited urgent attention, they were again reminded that ‘no serious attempt’ could be made at understanding or attempting to treat it until all researchers utilised the same ‘agreed requirements of clinical assessment’.^101^

## 3 Seeking a ‘Unified Approach’ to Clinical Assessment

These concerns led the co-ordinating committee to argue that their immediate priority was arranging a workshop where participants drew up plans for standard assessment methods to which all MRC-funded researchers ‘should conform’.^102^ Their belief that success in research hinged on adopting standard methods supports Keating and Cambrosio’s claim that one of the defining features of biomedicine after the Second World War has been the collective effort to ensure researchers across disciplines and institutions ‘achieve a common language’ when they document the symptoms or pathological markers associated with specific diseases.^103^ They argue this ‘will to standardise’ is not an ‘obstacle to fruitful work or innovation’, but is essential for the successful production, circulation and uptake of novel entities and findings.^104^ This view was held by the co-ordinating committee, who reassured the MRC they ‘did not have it in mind to “straitjacket” research’ and simply wanted to ‘ensure that studies should contain basic elements to allow findings from different centres to be usefully compared and pooled’.^105^

Although the MRC described the proposed workshop as an important ‘first step’ in co-ordinating research, Marsden’s committee soon found they were unable to organise it themselves thanks to their role as ‘collective referee’ for all applications concerning Alzheimer’s disease.^106^ Following a second meeting dedicated to reviewing applications, Marsden reminded committee members that their ‘ultimate purpose was coordination’ and claimed it ‘would be a pity if the opportunity to create a more unitary approach to research in the field were lost’.^107^ By December 1985, the co-ordinating committee decided to establish a steering committee whose sole job was to plan the workshop.^108^ Early in 1986, this new steering committee, chaired by the geriatrician Gordon Wilcock, identified 62 participants for the workshop, drawn from psychiatry, pathology, neurology, biochemistry, psychology, epidemiology, molecular biology and geriatric medicine, and asked each to specify what clinical information they believed was required to ‘(a) decide whether a person is suffering from dementia; (b) categorise the degree of dementia; and (c) decide on a clinical diagnosis of the cause of dementia’.^109^

The diversity in working practices was highlighted when respondents identified 36 different assessment schedules that were currently used to diagnose or measure the severity of dementia.^110^ In light of this ‘considerable variation’, the steering committee reiterated that the overriding aim of their workshop ‘should be to provide guidelines for the minimum data which should be collected in clinical and pathological studies on patients with presumed Alzheimer’s disease and dementia’.^111^ They drew up proposals for standard assessment guidelines and distributed them to all participants before the workshop, claiming their ‘guiding principles’ had been ‘firstly, that the proposals should be as brief as possible so as not to overburden research workers with an excess of data to be collected; and secondly, that the data to be collected should be specified as precisely as possible, so as to maximise the comparability between different research workers’.^112^ The proposals for clinical assessment comprised a list of questions for a friend or relative, followed by cognitive tests to establish ‘if the subject is impaired… and to give a measure of the severity’, and then a physical examination to ‘help avoid misdiagnosis of dementia’ by identifying any conditions that might impair performance on cognitive tests.^113^ To ensure they were ‘well evaluated’ and familiar to researchers, the cognitive tests incorporated all of the MMSE questions concerning orientation, object recognition, language comprehension and calculation, as well as the CAMDEX questions concerning abstract thought and historical events and figures.^114^

These draft proposals were accompanied by guidelines for the workshop, which the steering committee claimed had ‘not been organized like a conventional conference as it has a specific task to perform’.^115^ They instead modelled the workshop on the ‘consensus conferences’ which research councils increasingly used to produce guidelines for contentious procedures or disease classifications.^116^ When it was held in Oxford during September 1986, the workshop contained no formal presentations and each session centred on discussing a section of the proposal. Following a brief introduction, ‘aimed at explaining the reasons for the choices made in the relevant parts of the proposal’, participants highlighted any deficiencies and suggested alterations.^117^ The points made in each session were then incorporated into new guidelines that all participants reconsidered. Further alterations were made during the second day until the majority ‘agreed to a final version’.^118^

After this final draft was sent to all participants for written feedback, the co-ordinating committee approved the guidelines and they were published as a booklet by the MRC in 1987. This finalised schedule comprised guidelines for a semi-structured interview with a friend or relative of the subject, including questions about their psychiatric history, personality and evidence for cognitive impairment; a detailed cognitive examination of the subject, including MMSE questions concerning the time and location, language comprehension and basic arithmetic, and CAMDEX questions concerning abstract thought, recognition of household objects and famous individuals; and tests for physical difficulties that might impair test performance, such as deafness, visual handicap or involuntary tremors.

Like the American Psychiatric Association’s *Diagnostic and Statistical Manual of Mental Disorders*, then in its third edition,^119^ or the diagnostic criteria for Alzheimer’s disease developed at a 1984 consensus conference in the United States,^120^ the MRC guidelines were intended as both standards and *standardising*: imposing uniformity on what information was considered relevant and the means by which researchers collected it. The introduction to the published guidelines made this clear when it stated the MRC now expected all its funded researchers to utilise the guidelines ‘in order to achieve the purpose of comparing data collected in different studies’.^121^ While the MRC acknowledged it might allow exceptions, an applicant who did not use the guidelines to collect data was required to ‘explain why this is not appropriate’.^122^ A brief workshop report, which doubled as publicity for the published booklet, showed how the MRC’s aim of a ‘unified approach’ extended beyond their own projects when it expressed hopes ‘that others studying Alzheimer’s disease will also be willing to collect the same data’.^123^

The introduction to the published guidelines also stressed they were formulated specifically to obtain ‘recommended minimum data to aid comparison between research studies’ and were not to be used, as other tests had been, ‘to derive the clinical diagnosis of dementia or Alzheimer’s disease in an individual patient’.^124^ In response to a question asking whether the guidelines had ‘any meaning for diagnosis’, the steering committee claimed that while its various tests helped researchers ascertain ‘whether a person is demented’ and should be included in research, they did not ‘provide any rules by which one can go from the answers to the questions to diagnosis’.^125^ A longer introduction to the draft guidelines that were circulated for consultation argued this was because attempting to diagnose patients ‘may introduce too much rigidity too early in the research’.^126^ The focus of most research was expected to involve understanding Alzheimer’s disease at its early stages, while diagnostic criteria predominantly focussed ‘on the “core” of the condition rather than its boundaries’, imposing a cut-off that was ‘unhelpful for some research aims’.^127^ Using the MRC’s guidelines to ‘specify the information which should be collected, and the method by which it should be done’, the steering committee concluded, ‘does not impose a definition of the condition [and] allows for flexibility amongst research workers as to which subjects are included in a study’.^128^

This flexibility extended to the MRC’s guidelines themselves. Workshop participants had argued further research would alter ideas about which symptoms were considered significant for data collection, and the steering committee predicted their guidelines would be revised in two years.^129^ Like other groups who developed and sought to impose standard conventions for biomedical research,^130^ the steering committee viewed ‘regulation’ and ‘innovation’ as mutually constitutive: believing research on Alzheimer’s disease not only required standards in order to function but would reshape these standards once it functioned effectively.

## 4 The Politics of Standards: Criticising the MRC Guidelines

The steering committee’s readiness to alter their guidelines did not originate solely from a belief that research findings would change ideas about what data should be collected and compared. After receiving ‘substantive’^131^ criticisms from some workshop participants who read a final draft, the steering committee argued that revisions would also ‘have to take into account any feedback from workers in the field’.^132^ These criticisms and the steering committee’s responses highlight what Timmermans and Berg call the ‘politics of standardization’, where arguments for and against new conventions reflect an individual or group’s perspective on efforts to standardise working practices, including the consequences for professional freedom and ‘what configurations of things or people are brought into being’.^133^

Criticisms of the draft guidelines ranged from the general to the specific. Some workshop participants questioned whether research on Alzheimer’s disease even needed standard guidelines. Echoing concerns that standardisation weakened the autonomy of clinicians and researchers,^134^ the neurologist David Neary argued that ‘if one were forced too slavishly to adhere to questionnaires in order to obtain research funding from the MRC in the field of dementia, then this might inhibit original work’.^135^ Disputing the steering committee’s belief that standard guidelines would facilitate innovation, Neary warned that efforts to understand Alzheimer’s disease and similar conditions would be ‘retarded by research workers being held in the psychiatric chains of the questionnaire rather than carrying out their work in individual and original ways’.^136^ The psychiatrist John Copeland also portrayed standards as a form of restraint when he claimed that issuing guidelines under the ‘powerful imprimatur of the MRC’ meant the steering committee would ‘strait-jacket the methods which it intends researchers to use’.^137^ While he believed the MRC was ‘well within its remit to specify the areas of information and investigation which it would consider necessary for research projects’, Copeland doubted ‘whether it ought to try to specify the method by which this is done, i.e., in this instance, the form of the questions to be asked of subjects’.^138^

The steering committee dismissed this broad criticism and claimed workshop participants had overwhelmingly accepted the need for standard assessment criteria. The MRC guidelines, they continued, ‘will not inhibit original research’ and merely specified ‘the minimum clinical information which should normally be collected in research projects concerned with dementia’.^139^ The steering committee were more receptive to those who questioned certain aspects of the guidelines. They amended one memory test after several participants noted subjects were not prompted to repeat an address they had been given previously, and accepted Felicia Huppert’s offer to redraft sections that combined questions from the MMSE and the CAMDEX.^140^

Hinging on concerns about professional freedom and technical accuracy, these criticisms support Timmermans and Berg’s claim that ‘standards are inherently political because their construction and application transform the practices in which they become embedded’.^141^ This was also evident in criticisms that revealed a commitment to particular approaches and modes of analysis which researchers viewed as marginalised by the MRC’s standardising criteria. Even though the MRC had emphasised the need for ‘multidisciplinary cooperation’ in dementia research since the 1970s,^142^ its assessment guidelines for Alzheimer’s disease nevertheless drew on and prioritised the long-standing belief, held by the majority of British psychiatrists, neurologists and psychologists, that cognitive testing provided an ‘objectively calculable’ basis for differentiating patients and research subjects according to biological criteria.^143^ This presumption was critiqued by advocates of a more epidemiological outlook, who worked in the community rather than the clinic and sometimes viewed themselves as ‘divergent from mainstream medicine’.^144^ The epidemiologist Carole Brayne, for instance, claimed the draft guidelines only asked researchers to test for physical factors that might impair test performance and provided ‘no question asking whether the subject can read or write… or their first language’.^145^ The steering committee, again, were receptive to this criticism and added a section in the published guidelines asking the researcher to note down the ‘educational level attained’ by the subject before undertaking the cognitive assessment.^146^

Brayne was not the only workshop participant to claim that non-medical factors might affect how subjects performed during cognitive assessments. The issue was raised in more detail by the émigré psychiatrist Klaus Bergmann, who worked at the Maudsley Hospital in London and had previously worked with Martin Roth and colleagues in Newcastle upon Tyne, where he used a series of tests and semi-structured interviews to determine the relative frequencies of psychiatric illnesses in a community sample during the 1970s.^147^ Bergmann’s correspondence illustrates how debates about standards do not simply reflect professional considerations but can also embody broader socio-political concerns, with both existing ‘in a continuous and emerging interactive relationship’.^148^ After attending the workshop and receiving a final draft of the guidelines, Bergmann wrote to the steering committee and argued that asking patients to identify historical figures during memory tests embodied ‘the usual middle class assumption that everyone knows about General de Gaulle and Neville Chamberlain’.^149^ Bergmann warned that ‘ill-educated and hesitant working class patients will register falsely on this’, leading researchers to mistakenly believe they had Alzheimer’s disease, and urged the steering committee to delete the questions.^150^

Bergmann was one of several psychiatrists who worked in the community and adopted an epidemiological perspective during the 1960s and 1970s: arguing that social factors sometimes determined how patients responded to cognitive tests, and that a relatively poor outcome did not always mean an individual was suffering from psychiatric disease. This possibility was first raised following work that sought to assess rates of psychiatric disease among elderly residents of Newcastle upon Tyne in the 1960s. Describing their initial results in 1964, David Kay, Paul Beamish and Martin Roth admitted that judgements in mild or moderate cases were ‘unavoidably arbitrary’,^151^ despite the use of cognitive tests, and when Kay followed up the cohort several years later he discovered that many of those initially thought to have dementia showed no sign of deterioration.^152^ This finding led figures at the DHSS to question whether ‘the numbers derived from the Newcastle study represent substantial exaggerations of the true position’ and to argue further work was needed to clarify these ‘problems of definition and interpretation’.^153^

Shortly after Bergmann arrived in Newcastle, the DHSS provided funding for a project in which he worked with David Kay and the psychologists Paul Britton and Eleanor Foster to assess survivors from the original cohort over a period of seven years. In analysing the relationship between test scores and outcome, ie. survival or death, they considered variables such as age, sex, rating of physical disability and the formal measure of class first developed by the Registrar General’s Office in 1911.^154^ Initially designed to explain the differential health outcomes of various social groups, and specifically why the poor were more subject to illness, the Registrar General’s schema categorised individuals and households according to the following occupational hierarchy: (i) professional occupations such as doctors and the clergy; (ii) intermediate occupations such as teachers and administrative workers; (iii) non-manual skilled workers such as cashiers, and manual skilled workers such as plumbers; (iv) semi-skilled occupations such as waitresses; and (v) unskilled occupations such as labourers or cleaners.^155^ In a 1977 paper, Kay, Britton, Bergmann and Foster outlined how they found a high correlation between poor test scores and mortality among individuals in the first three of the General Registrar’s social classes, with most low-scoring individuals dying two years after their first assessment, but notably claimed they found ‘no difference in mortality between high and low scoring subjects’ in the last two groups of social classes.^156^

With only eight per cent of low-scoring individuals dying in social groups four and five, compared to fifty-six per cent in groups one to three, Bergmann and colleagues argued that poor test scores here should be interpreted as ‘merely the tail of the distribution of IQs in the general population, rather than deterioration from a previously higher level’.^157^ While they maintained that cognitive tests remained useful for measuring intellectual function in older people, they argued these findings highlighted ‘certain problems in interpreting the results… among people of poor educational level or low basic intelligence’.^158^ This led them to conclude that existing methods did ‘not appear to distinguish reliably between the mentally dull or mildly retarded, and the mentally deteriorated old person’, posing significant problems for the recruitment of research subjects and ‘the administration and planning of services’.^159^

Throughout this analysis, Bergmann and colleagues conflated an individual’s position in a socio-occupational hierarchy with their perceived educational ability, but they were not alone in doing so. Their conclusions echoed officials at the government’s Office of Population Censuses and Surveys, who maintained the Registrar General’s index and claimed in 1970 that its grading of social class was ‘naturally correlated with, and its application conditioned by, other factors such as education and economic environment’.^160^ The association was furthered in 1980, when Martin Roth delivered a lecture on ‘senile dementia and its borderlands’ to the American Psychopathological Association. Roth argued ‘poor intellectual performance’ was increasingly known to cause problems for those who used cognitive tests to identify mild or moderate cases of dementia.^161^ These problematic cases, he continued, were ‘drawn mainly from the lower social strata in the community, handicapped by poor social skills and limited intelligence’.^162^ Roth used the growing concern over ‘false positives’ to stress the need for clinical acumen. Cognitive tests, he warned, merely provided ‘a valuable adjunct in assessment of patients suspected of dementia’, and ‘no psychological measure should be allowed to override a judgement derived from careful history-taking and thorough clinical examination’.^163^

Other researchers who worked in Newcastle upon Tyne placed less emphasis on clinical judgement and argued ‘further improvements should be made to develop acceptable tests which discriminate between groups of old people who have very different life prognoses’.^164^ David Kay, for one, believed the fault lay with tests concerning historical figures and dates that Roth had used since the 1950s, which he claimed made it difficult for researchers to distinguish ‘subjects with low intelligence or poor education from those with brain syndromes’.^165^ Garry Blessed also believed that questions asking patients to recall the dates of historical events and figures had ‘more to do with education than brain function’.^166^ When he designed his own dementia scale in the mid-1960s, Blessed substituted these for questions concerning ‘items of personal memory’, such as the patient’s birthday and address, which he claimed effectively discriminated between individuals with dementia and those whose responses may be conditioned by social factors.^167^

Klaus Bergmann urged the steering committee to do likewise in 1986 and replace its ‘middle class biased’ memory tests with questions that ‘asked after the patient’s age and their birthday’.^168^ The steering committee had, in fact, already sought advice on whether to include a test for ‘famous names’ before they received Bergmann’s criticism.^169^ The psychologist Stuart Hamilton, from the University of Manchester’s Age and Cognitive Performance Unit, wrote to assure them that a famous names test had been validated in 1978 to assess recent and remote memory: by asking subjects to identify contemporary figures, such as the peer and suspected murderer Lord Lucan, and others who were famous during the early twentieth century, such as the musical hall entertainer Florrie Forde.^170^ Hamilton told the steering committee that all two hundred subjects tested recognised ten ‘very famous’ names, including President General de Gaulle and Neville Chamberlain, and advised them that failure to recognise any of the very famous names and two or more other names should be considered ‘indicative of a severe remote memory failure’.^171^

This information persuaded the steering committee to overlook Bergmann’s criticism. Their finalised guidelines included a test for recent memory that asked subjects to name the current Prime Minister and President of the United States, and a test for remote memory that asked them to identify Neville Chamberlain and the Cambridge spy Guy Burgess. Rejecting Bergmann’s claim that these tests discriminated against ‘ill educated’ patients, the steering committee argued they ‘had been included on the basis of research work done and the advice of specialists in the field’, and noted several participants at the workshop ‘thought that a test of famous names should be included’.^172^

Once the guidelines were published, it became clear that few of those who designed them would be receiving feedback or undertaking revisions in future. The steering committee was disbanded early in 1987, although Gordon Wilcock ‘expressed willingness’ to review the guidelines if needed;^173^ later that year, members of Marsden’s committee argued they had fulfilled their remit by helping ‘to co-ordinate research into Alzheimer’s disease by producing guidelines for assessment’ and reviewing ‘several major grant applications’.^174^ During their fifth and final meeting in May 1987, the co-ordinating committee recommended they ‘should be disbanded’ as well.^175^ After discussing possible treatment of Alzheimer’s disease with a drug that aimed to boost acetylcholine levels, the co-ordinating committee predicted ‘clinical trials… are likely to become increasingly important over the coming years’. Their final act was to recommend that the MRC establish another committee to ‘set up the framework required’ for drug testing and advise on ‘assessment methods to be used, likely rates of attrition, etc’.^176^

By encouraging a new committee that prioritised clinical trials, however, the co-ordinating committee helped undermine their goal of a ‘unified approach’ to assessment and data collection in research. When members of this new Alzheimer’s disease Trials Committee circulated a working paper in 1988, they claimed the MRC’s guidelines were useful for ‘setting out the minimum data to be collected’ but argued ‘more comprehensive assessments would be needed for particular studies’.^177^ ‘Methods of assessing mental state currently in use were designed, not so much for measuring change, or rate of change in response to a treatment, as in quantifying the degree of mental defect present at the time of diagnosis’, they outlined, whereas ‘since any drug trial is likely to be looking at improvements in memory and function, specific validated assessments are needed in these areas’. Committee members also predicted a series of new tests would be needed to monitor change in different areas, such as intellectual performance and quality of life, and across the various groups recruited for clinical trials, including seriously ill patients in hospitals and ‘less severely disabled’ subjects drawn from the community.^178^

At the same time, more researchers began to publicly argue that no single assessment or set of guidelines effectively demarcated Alzheimer’s disease from normal ageing. In 1988 the neurologist Carole Brayne and the psychiatrist Paul Calloway claimed tests undertaken on 400 elderly individuals in Cambridgeshire revealed no clear distinction. Even though their assessment schedule ‘covered all areas suggested by the MRC Working Group’, Brayne and Calloway argued that ‘in a truly representative community sample there is a continuous distribution of at least some of the manifest variables associated with Alzheimer’s disease’.^179^ They concluded that distinguishing between normal ageing and cases of Alzheimer’s disease remained ‘arbitrary’, despite recent efforts to improve and standardise cognitive tests.^180^

Similar claims appeared in an article by the psychologist Adrienne Little and colleagues, who documented attempts to classify residents of a London care home as ‘diseased’ or ‘normal’ using assessments of self-care ability and intellectual function.^181^ Little and colleagues argued that while many individuals classified as ‘diseased’ performed significantly worse during initial testing and follow-ups than those classified as ‘normal’, there was again ‘substantial overlap between groups for all measures, with the ranges of scores of the two groups virtually coinciding’.^182^ They partly attributed this overlap to the fact they used a test ‘developed and validated for use with a different population (elderly living at home)’.^183^ But they also attributed it to individuals who were initially classified as ‘diseased’ but showed no evidence of cognitive decline over the following two years. Designating certain tests as standards that could be used in any setting and on all individuals, they concluded, ‘would produce many errors of classification’.^184^ Perhaps influenced by Klaus Bergmann, who they thanked for ‘support and guidance’, Little and colleagues argued that elderly subjects would only be classified into ‘more distinct groups’ once researchers developed specific tests for different populations and, crucially, recognised ‘the importance of tailoring assessments to the ability of subjects when monitoring change’.^185^

While these articles attributed classificatory errors to a lack of specificity in cognitive tests, the writer of an anonymous piece for *The Lancet* went further when seeking to explain the ‘preponderence of false positives’.^186^ They argued the symptoms associated with Alzheimer’s disease mirrored a general ‘loss of adaptability’ in ageing, and that each case was designated as normal or diseased according ‘to the perceptions, personality, and functioning of the affected individual, his family, and society’. But these decisions reflected preconceived ideas about educational ability and social class: ‘Thus a professor with early dementia may be classified as absent minded; a labourer diseased’.^187^ The author stated that demarcating Alzheimer’s disease from normal ageing could never be standardised, and it was ‘important for politicians and other fund givers as well as elderly people to understand these issues’.^188^

None of these critiques questioned the biological basis of Alzheimer’s disease or sought to revive psychodynamic claims that it originated from inability to cope with social pressures, but instead took issue with the belief that standard assessment guidelines enabled researchers to distinguish all cases of Alzheimer’s disease from normal ageing. This argument clearly resonated with many of the researchers who worked on the condition from the 1980s onwards. The authors of a 2002 review claimed no single test could be expected to screen different populations, assess the severity of symptoms and monitor change in ‘major clinical domains’ such as mood, cognition or quality of life.^189^ Researchers and clinicians thus had to choose from a ‘multitude’ of assessments that, while posing a ‘formidable challenge… in deciding which is most appropriate’, guarded them against ‘making the wrong choice’ and using a test not specifically developed for and validated on their chosen population or symptom.^190^ In contrast to those who previously argued that particular assessment schedules should be adopted as ‘agreed standards’ by researchers, these authors concluded that ‘the ideal scale does not exist’.^191^

Tellingly, this review also suggested that numerical cut-offs in tests, which indicated when someone was likely to have a condition such as Alzheimer’s disease, should be constantly adjusted to account for variables such as ‘lack of education’.^192^ While they no longer conflate education and class, as Bergmann, Kay and others did in the 1970s and 1980s, the researchers who endorse these changes still question the extent to which tests enable researchers and clinicians to ‘differentiate according to nature and not to prejudice’.^193^ By arguing that biological factors cannot solely explain the conduct of elderly subjects in cognitive tests, they challenge both the ‘will to standardise’ dementia research and the presumption that tests provide unmediated access to a psychiatric, psychological or biochemical ‘reality’ that exists independently of the social conditions under which it is investigated.^194^ Despite the overriding focus on routine assessments and biomedical theories of causation, researchers argued, and continue to argue, that the methods used to demarcate Alzheimer’s disease from normal ageing are most effective if investigators are attuned to the possible influence of social factors. Identifying cognitive decline in face-to-face encounters with research subjects or patients, still essential for treatment and research, remains a complex process that defies standardisation and cannot be decontextualised from everyday life.

## 5 Conclusions

A growing number of historians and sociologists portray standard diagnostic manuals and assessment guidelines as critical nodes in the assemblage of interacting disciplines, practices, theories, institutions and bureaucracies that constitute modern biomedicine.^195^ For Martyn Pickersgill they are among ‘the most important means by which professional power operates’: structuring the methods clinicians and researchers use to categorise symptoms and transforming new groups of people into objects of scientific inquiry.^196^ Several of these accounts also show that researchers oppose standards on the grounds they threaten professional autonomy, undermine particular ways of working and risk ‘medicalising normality’ by arbitrarily distinguishing health from disease.^197^ They demonstrate how this criticism ensures that standardisation is a dynamic process, in which assessment guidelines are constantly updated. A smaller but no less important body of work also shows how differing contexts and cultural norms ensure that these debates vary across geographical locations, ‘along with the material practices used to define a given disease entity’.^198^

These latter studies caution us against locating debates about standardisation solely within the ‘regulatory-political environment from which standards emerge’.^199^ Isolating them from social contexts ignores the extent to which ‘ “science” and “society” mutually constitute and legitimate one another’.^200^ As Raymond Williams argued in *The Long Revolution*, anticipating the idiom of ‘co-production’ now popular in science and technology studies,^201^ activities in science, politics, art and religion belong in a ‘world of active and interacting relationships, which is our common associative life’, and we can never fully understand ideas or practices in any of these domains until we ‘relate our studies… to the actual and complex organisation’.^202^ This broad perspective is vital to appreciating the significance of debates about standard assessment guidelines for Alzheimer’s disease in the 1980s. The MRC sought to impose standard guidelines for scientific reasons, to facilitate multi-centre research by ensuring investigators collected data that could be shared across projects; and some criticism from researchers hinged on professional concerns that standards restrained autonomy and favoured certain modes of analysis. But other criticism reflected a belief that standard memory tests were problematic because non-biomedical issues such as educational ability and social background influenced the conduct of research subjects and potentially distorted test outcomes. While their intention was to revise guidelines so that Alzheimer’s disease could be more precisely identified, researchers who made this argument problematised specific memory tests *and* those ‘mentally dull’ individuals who needed to be clearly demarcated from genuine cases of ‘mental deterioration’.^203^ In doing so, and by arguing that these cases always came from the ‘lower strata’ of society, they ultimately drew on and perpetuated a long-standing British concern with identifying factors that distinguished the professional middle classes from the semi-skilled or unskilled working classes.^204^

Researchers no longer emphasised class by the 1990s, but they still asserted the importance of non-medical factors by maintaining that tests needed adjusting to account for an individual’s educational background. While the problematic variables researchers identify change over time, this enduring critique of ‘the will to standardise’ demonstrates how they perennially encounter Alzheimer’s disease not just as a ‘problem of brains in labs, but of human beings in time, space, culture and history’.^205^ And this helps us reposition the elderly subjects of research as critical figures in the recent history of Alzheimer’s disease, which at present focusses on biomedical scientists and competing theories of disease causation. While patients and research subjects had no say in developing the MRC guidelines, their differing backgrounds and responses to tests nevertheless played a major role in shaping research methods, by ensuring that researchers and funding bodies continued to have trouble distinguishing normal from abnormal in old age. As Williams and others argue, ideas about class, education and social relations profoundly influence the activities that make up our ‘common associative life’, and differentiating between ‘senile dementia and its borderlands’ was clearly no exception.

## Footnotes

1.

German E. Berrios , A History of Mental Symptoms (Oxford: Oxford University Press, 1999), 195–196.

2.

Thomas G. Beach , ‘The History of Alzheimer’s Disease: Three Debates’, Journal of the History of Medicine and Allied Sciences, 42, 3 (1987), 327–349. I have put the term ‘normal’ ageing in quotes here because old age psychiatrists and geriatricians have rarely held consensus on what normal ageing is and how it can be measured; for more detail, see Margaret Lock, *The Alzheimer Conundrum: Entanglements of Dementia and Aging* (Princeton: Princeton University Press, 2013), 42–4.3305698

3.

Anon, ‘Dementia – The Quiet Epidemic’, British Medical Journal, 1(1978), 1–2.620128PMC1602470

4.
Beach, *op. cit.* (note 2), 328.

5.
David Cameron, Transcript of Speech to the 2012 Dementia Conference, available online at https://www.gov.uk/government/speeches/transcript-prime-ministers-speech-to-the-dementia-2012-conference.

6.

Jesse F. Ballenger , Self, Senility and Alzheimer’s Disease in Modern America: A History (Baltimore, MD: Baltimore University Press, 2006), 91–93.

7.

*Ibid.*, 81–6; Duncan Wilson, ‘Quantifying the Quiet Epidemic: Diagnosing Dementia in late Twentieth-Century Britain’, *History of the Human Sciences*, 27, 5 (2014), 126–46.10.1177/0952695114536715PMC436147025866448

8.

Tiago Moreira , ‘Truth and hope in drug development and evaluation in Alzheimer’s disease’, in Jesse F. Ballenger , Peter J. Whitehouse , Constantine G. Lyketsos , Peter V. Rabins and Jason H. T. Karlawish (eds), Treating Dementia: Do We Have a Pill for It? (Baltimore, MD: Johns Hopkins University Press, 2009), 211–230.

9.
Ballenger, *op. cit.* (note 6), 113–51; Patrick J. Fox, ‘The role of the concept of Alzheimer’s Disease in the development of the Alzheimer’s Association in the United States’, in Peter J. Whitehouse, Konrad Maurer and Jesse F. Ballenger (eds), *Concepts of Alzheimer’s Disease: Biological, Clinical and Historical Perspectives* (Baltimore and London: Johns Hopkins University Press, 2000), 209–33.

10.
Moreira, *op. cit.* (note 8), 218.

11.
Ballenger, *op. cit.* (note 6), 111.

12.

Rudolph E. Tanzi and Ann B. Parson , Decoding Darkness: The Search for the Genetic Causes of Alzheimer’s Disease (Cambridge, MA: Perseus Publishing, 2000), xiv.

13.

*Ibid.*; see also Katzman and Bick, ‘The rediscovery of Alzheimer’s Disease in the 1960s and 1970s’, in Whitehouse, Maurer and Ballenger, *op. cit.* (note 9), 104–14.

14.
For more historical background, see Joan Austoker and Linda Bryder (eds), *Historical Perspectives on the Role of the MRC: Essays in the History of the Medical Research Council in the United Kingdom and its Predecessor, the Medical Research Committee, 1913–1951* (Oxford and New York: Oxford University Press, 1989).

15.

Charles E. Rosenberg , ‘The Tyranny of Diagnosis: Specific Entities and Individual Experience’, The Millbank Quarterly, 80, 2 (2002), 237–260, on 255.10.1111/1468-0009.t01-1-00003PMC269011012101872

16.
Anon, Report of the Second Meeting of the Co-ordinating Committee (20 May 1985). Held at the National Archives, London (henceforth National Archives), FD23/2290.

17.
Medical Research Council, *Report from the MRC Alzheimer’s Disease Workshop* (London: Medical Research Council, 1987), 1.

18.

Rebecca Schilling and Stephen T. Casper , ‘Of Psychometric Means: Starke R. Hathaway and the Popularization of the Minnesota Multiphasic Personality Inventory’, Science in Context, 28, 1 (2015), 77–98: 80.2583257110.1017/S0269889714000337

19.
Nikolas Rose, *Inventing Ourselves: Psychology, Personhood and Power* (Cambridge: Cambridge University Press, 1998), 87. See also Schilling and Casper, *ibid*; Wilson, *op. cit.* (note 7); Kurt Danziger, *Constructing the Subject: Historical Origins of Psychological Research* (Cambridge: Cambridge University Press, 1994).

20.
Rose, *ibid.*, 57.

21.
On biomedicine see Adele E. Clarke , Janet K. Shim , Laura Mamo , Jennifer R. Fosket and Jennifer R. Fishman , ‘Biomedicalization: Technoscientific Transformations of Health, Illness and US Biomedicine’, American Sociological Review, 68, 2 (2003), 161–194.

22.
Peter Keating and Alberto Cambrosio, *Biomedical Platforms: Realigning the Normal and Pathological in Late-Twentieth-Century Medicine* (Cambridge, MA: MIT Press, 2006), 305. See also Lucie Gerber and Jean-Paul Gaudillière, ‘Marketing Masked Depression: Physicians, Pharmaceutical Firms and Redefinition of Mood Disorders in the 1960s and 1970s’, *Bulletin of the History of Medicine*, 90 (2016), 455–90; Alberto Cambrosio, Peter Keating, Thomas Schlich and George Weisz, ‘Regulatory Objectivity and the Generation and Management of Evidence in Medicine’, *Social Science and Medicine*, 63, 1 (2006), 189–99; Stefan Timmermans and Marc Berg, *The Gold Standard: The Challenge of Evidence-Based Medicine and Standardization in Health Care* (Philadelphia: Temple University Press, 2003).

23.
Rose, *op. cit.* (note 19), 111–13.

24.
Danziger, *op. cit.* (note 19), 186.

25.
Nikolas Rose and Joelle M. Abi-Rached, *Neuro: The New Brain Sciences and the Management of the Mind* (Princeton and Oxford: Princeton University Press, 2013), 10. On psychodynamic approaches to dementia in the early twentieth century see Ballenger, *op. cit.* (note 6), 36–56.

26.
Danziger, *op. cit.* (note 19), 168.

27.

*Ibid.*, 174.

28.
Klaus Bergmann to Megan Davies (20 November 1986), National Archives, FD23/2300.

29.

Tiago Moriera , Carl May and John Bond , ‘Regulatory Objectivity in Action: Mild Cognitive Impairment and the Collective Production of Uncertainty’, Social Studies of Science, 39, 5 (2009), 664–690: 666.

30.
Timmermans and Berg, *op. cit.* (note 22), 22.

31.
Martin Roth, ‘Problems of an Ageing Population’, *British Medical Journal*, 1 (1960), 1226–30: 1226. See also International Association of Gerontology, *Old Age in the Modern World, Report of the Third Congress of the International Association of Gerontology, London, 1954* (Edinburgh and London: E. & S. Livingstone, Ltd., 1955).

32.
Roth, *ibid.*, 1226; Aubrey Lewis, ‘Ageing and Senility: A Major Problem of Psychiatry’, *Journal of Mental Science*, 92 (1946), 150–70: 169.10.1192/bjp.92.386.15020984301

33.
Roth, *op. cit.* (note 31), 1227, 1229.

34.
For more detail see Wilson, *op. cit.* (note 7), 130–3.

35.

Martin Roth and John D. Morrisey , ‘Problems in the Diagnosis and Classification of Mental Disorder in Old Age; with a Study of Case Material’, Journal of Mental Science, 98 (1952), 64–80:71.10.1192/bjp.98.410.6614898208

36.

Martin Roth and Barbara Hopkins , ‘Psychological Test Performance in Patients Over Sixty. I. Senile Psychoses and Affective Disorders of Old Age’, Journal of Mental Science, 99 (1953), 439–450: 449.1306998910.1192/bjp.99.416.439

37.
Wilson, *op. cit.* (note 7), 131. On mental illnesses as natural kinds, see Rachel Cooper, *Psychiatry and Philosophy of Science* (Stocksfield: Acumen Publishing, 2006).

38.
On how the 1959 Mental Health Act endorsed community care over institutional treatment, see Hugh Freeman , ‘Psychiatry in the National Health Service, 1948–1998’, British Journal of Psychiatry, 175 (1999), 3–11.1062176110.1192/bjp.175.1.3

39.

Martin Roth and D.H. Myers , ‘The Diagnosis of Dementia’, British Journal of Hospital Medicine, 2 (1969), 705–717: 715.

40.
W. McAdam and R.A. Robinson, ‘Senile Intellectual Deterioration and the Electroencephalogram: A Quantitative Correlation’, *British Journal of Psychiatry*, 102 (1956), 819–25; Garry Blessed, Bernard E. Tomlinson and Martin Roth, ‘The Association Between Quantitative Measures of Dementia and of Senile Change in the Cerebral Grey Matter of Elderly Subjects’, *British Journal of Psychiatry*, 114 (1968), 797–811.

41.
Wilson, *op. cit.* (note 7), 134–5; Claire Hilton (ed.), *The Development of Old Age Psychiatry from the 1960s until 1989* (University of Glasgow: Guthrie Trust, 2008), 14.

42.
Garry Blessed, interview with the author (Ponteland, Northumbria, September 2013).

43.
Blessed, Tomlinson and Roth, *op. cit.* (note 40), 805.

44.
Martin Roth, ‘Classification and aetiology of mental disorders of old age: some recent developments’, in David W. Kay and Alexander Walk (eds), *Recent Developments in Psychogeriatrics: A Symposium* (London: Headley), 1–17, on 2; for more detail on the background and impact of this research, see Wilson, *op. cit.* (note 7).

45.
Bernard E. Tomlinson, Garry Blessed and Martin Roth, ‘Observations on the Brains of Demented Old People’, *Journal of Neurological Science*, 11 (1970), 243–35; David W.K. Kay, Paul Beamish and Martin Roth, ‘Old Age Mental Disorder in Newcastle upon Tyne. Part 1: A Study of Prevalence’, *British Journal of Psychiatry*, 110 (1964), 146–58.10.1192/bjp.110.465.14614130463

46.
See Wilson, *op. cit.* (note 7), 134.

47.
Psychiatry Committee Policy Review (1975), National Archives, FD23/4388.

48.
Anon, Membership of the Committee (1976), National Archives, FD23/4388; W.A. Lishman, *Organic Psychiatry: The Psychological Consequences of Cerebral Disorder* (Oxford: Blackwell, 1978). On why psychodynamic approaches failed to gain a foothold in Britain, see Freeman, *op. cit.* (note 38).

49.
Anon, *ibid*.

50.

W.A. Lishman , Senile and Presenile Dementias: A Report of the MRC Subcommittee (London: Medical Research Council, 1977), 23.

51.
W. A. Lishman, Draft Report of the Subcommittee (1976), II, National Archives FD23/4388.

52.

*Ibid*.

53.

*Ibid*.

54.

*Ibid*.

55.

*Ibid*.

56.

*Ibid*.

57.

*Ibid*.

58.
Anon, Minutes of the Seventh Meeting of the Psychiatry Committee (March 1976), National Archives, FD23/4388.

59.

*Ibid*.

60.

*Ibid*.

61.

*Ibid*.

62.
Lishman, *op. cit.* (note 50), 14.

63.

*Ibid*.

64.

*Ibid.*, 19–20.

65.
Psychiatry Committee Policy Review (1977), National Archives, FD10/332.

66.
Anon, ‘Call for Dementia Research’, *Nature*, 269 (1977), 645.

67.

Elaine Perry , Robert Perry , Garry Blessed and Bernard Tomlinson , ‘Necroscopy of Central Cholinergic Deficits in Senile Dementia’, The Lancet, 310 (1977), 189.10.1016/s0140-6736(77)91780-964712

68.

Anon, ‘Notes and News: MRC’s View of Dementia’, The Lancet, 310(1977), 1191.

69.

*Ibid*.

70.
Alwyn Lishman to Katherine Levy (12 December 1977), National Archives, FD23/4388.

71.
Lishman, *op. cit.* (note 51).

72.
Martin Roth, Application for a Project Grant: Multidisciplinary Investigations into Senile Dementia (January 1978), National Archives, FD10/447.

73.

*Ibid*.

74.

*Ibid*.

75.

*Ibid*.

76.

*Ibid*.

77.
Anon, Note for File (January 1978), National Archives, FD10/447.

78.

M. Roth , C. Tym , C.Q. Mountjoy , F.A. Huppert , H. Hendrie , S. Verma and R. Goddard , ‘CAMDEX: A Standardised Instrument for the Diagnosis of Mental Disorder in the Elderly with Special Reference to the Early Detection of Dementia’, British Journal of Psychiatry, 49, 6 (1986), 698–709, on 698.10.1192/bjp.149.6.6983790869

79.

Martin Roth , Felicia Huppert , C.Q. Mountjoy and Elizabeth Tym , CAMDEX-R: The Cambridge Examination for Mental Disorders of the Elderly (Cambridge and New York: Cambridge University Press, 1998), 25–35.

80.
Marshal F. Folstein, Susan E. Folstein and Paul R. McHugh, ‘ “Mini-Mental State”: A Practical Method for Gauging the Cognitive State of Patients for the Clinician’, *Journal of Psychiatric Research*, 12 (1975), 189–98.10.1016/0022-3956(75)90026-61202204

81.
Felicia A. Huppert, Application for a Project Grant: ‘Clinical and Cognitive Investigations of the Early Stages of Dementia in the Elderly’ (April 1985), National Archives, FD23/2289.

82.

*Ibid*.

83.
Roth *et al.*, *op. cit.* (note 79).

84.
Roth and Myers, *op. cit.* (note 39), 715.

85.
Huppert, *op. cit.* (note 81).

86.

*Ibid*.

87.
Anon, Comments on Huppert, Clinical and Cognitive Investigations of the Early Stages of Dementia in the Elderly (1985), National Archives, FD23/2289.

88.
Raymond Levy and Robin Jacoby, Application for a Project Grant: ‘A Longitudinal Study of the Clinical Picture and Outcome of Senile Dementia of the Alzheimer Type’ (April 1985), National Archives, FD23/2289.

89.
Anon, Comments on Levy and Jacoby (1985), National Archives, FD23/2289. Emphasis in original.

90.
Anon, MRC Co-ordinating Committee: Terms of Reference (April 1985), National Archives, FD23/2289.

91.

*Ibid*.

92.
Anon, Co-ordinating Committee: List of Members (April 1985), National Archives, FD23/2289.

93.
Lishman, *op. cit.* (note 50), 1.

94.
Ballenger, *op. cit.* (note 6), 89.

95.
Anon, MRC Co-ordinating Committee on Alzheimer’s Disease, Report of First Meeting (16 April 1985), National Archives, FD23/2289.

96.

*Ibid*.

97.

*Ibid*.

98.

*Ibid*.

99.
Roth and Myers, *op. cit.* (note 39), 715. See also J.E. Cooper, R.E. Kendall, B.J. Gurland, L. Sharpe, J.R.M. Copeland and R. Simon, *Psychiatric Diagnosis in New York and London* (London and New York: Oxford University Press, 1972).

100.
Huppert, *op. cit.* (note 81).

101.
Anon, *op. cit.* (note 95).

102.

*Ibid*.

103.
Keating and Cambrosio, *op. cit.* (note 22), 333.

104.

*Ibid.*, 331–2.

105.
Anon, *op. cit.* (note 95).

106.
Anon, ‘Subcommittee on Alzheimer’s Disease’, *MRC News* (September 1985), National Archives, FD23/2294.

107.
Anon, Minutes of Second Meeting (16 May 1985), National Archives, FD23/2290.

108.
Anon, Minutes of Third Meeting (3 December 1985), National Archives, FD23/2294.

109.
Anon, Background to Workshop (December 1986), National Archives, FD23/2300.

110.
Anon, Report of the Second Meeting of the Steering Committee (16 May 1986), National Archives, FD23/2292.

111.

*Ibid*.

112.
The MRC Alzheimer’s Disease Workshop Steering Committee, Recommended Minimum Data to be Collected in Research Studies on Alzheimer’s Disease (1986), National Archives, FD23/2301.10.1136/jnnp.52.6.693PMC10320182664087

113.
Anon, Commentary on Clinical Assessment (September 1986), National Archives, FD23/2292.

114.

*Ibid*.

115.
Anon, Introduction to the Workshop (September 1986), National Archives, FD23/2292.

116.
See Keating and Cambrosio, *op. cit.* (note 22), 316–20.

117.
Anon, *op. cit.* (note 115).

118.
The MRC Alzheimer’s Disease Workshop Steering Committee, *op. cit.* (note 112).

119.
See Hannah S. Decker , The Making of DSM III: A Diagnostic Manual’s Conquest of American Psychiatry (Oxford and New York: Oxford University Press, 2013).

120.
See Moriera, *op. cit.* (note 8), 215; Guy McKhann, David Drachman, Marshall Folstein, Robert Katzman, Donald Price and Emanuel M. Stadlan, ‘Clinical Diagnosis of Alzheimer’s Disease: Report of the NINCDS-ADRDA Work Group Under the Auspices of the Department of Health and Human Services Task Force on Alzheimer’s Disease’, *Neurology*, 34 (1984) 939–44.10.1212/wnl.34.7.9396610841

121.
Medical Research Council, *op. cit.* (note 17), 4.

122.

*Ibid*.

123.
Anon, Report from the MRC’s Alzheimer’s Disease Workshop (1987), National Archives, FD23/2297.

124.
Medical Research Council, *op. cit.* (note 17), 1.

125.
Anon, Summary of Points Made (1987), FD23/2301.

126.
The MRC Alzheimer’s Disease Workshop Steering Committee, *op. cit.* (note 112).

127.

*Ibid*.

128.

*Ibid*.

129.

*Ibid*.

130.
See Keating and Cambrosio, *op. cit.* (note 22), 259.

131.
Megan Davies to Gordon Wilcock (3 December 1986), National Archives, FD23/2300.

132.
Anon, Report of the Fifth Meeting of the MRC Co-ordinating Committee (5 May 1987), National Archives, FD23/2297.

133.
Timmermans and Berg, *op. cit.* (note 22), 22–3. On how clinical guidelines and protocols ‘make up’ new groups of patients, see Ian Hacking, ‘Lost in the Forest’, *London Review of Books* (8 August 2013), accessed online at http://www.lrb.co.uk/v35/n15/ian-hacking/lost-in-the-forest; *Idem*, ‘Making up people’, in Thomas C. Heller, Morton Sosna and David E. Wellbery (eds), *Reconstructing Individualism: Autonomy, Individuality and the Self in Western Thought* (Stanford: Stanford University Press, 1986), 160–71.

134.
See Timmermans and Berg, *op. cit.* (note 22), 84–6.

135.
David Neary to Megan Davies (28 November 1986), National Archives, FD23/2300.

136.

*Ibid*.

137.
John Copeland to Megan Davies (16 December 1986), National Archives, FD23/2300.

138.

*Ibid*.

139.
Anon, *op. cit.* (note 125).

140.

*Ibid*.

141.
Timmermans and Berg, *op. cit.* (note 22), 22.

142.
Lishman, *op. cit.* (note 50), 9.

143.
Rose, *op. cit.* (note 19), 90.

144.
Hilton, *op. cit.* (note 41), 16.

145.
Carol Brayne to Megan Davies (27 November 1986), National Archives, FD23/2300.

146.
Medical Research Council, *op. cit.* (note 17), 6.

147.
Hilton, *op. cit.* (note 41), 37–40.

148.

Tiago Moriera , ‘Health Care Standards and the Politics of Singularities: Shifting In and Out of Context’, Science, Technology and Human Values, 37, 4 (2012), 307–331: 308.

149.
Bergmann to Davies, *op. cit.* (note 28).

150.

*Ibid*.

151.
Kay, Beamish and Roth, *op. cit.* (note 45), 151.

152.

David W.K. Kay , ‘Interview with Katherine Bick’, in Robert Katzman and Katherine Bick (eds), Alzheimer’s Disease: The Changing View (London: Academic Press, 2000), 237–251: 246–7.

153.
B.A. Harrison, Proposed New Study of the Elderly Mentally Disordered in Newcastle upon Tyne (February 1973), National Archives, MH166/1304.

154.

D.W.K. Kay , P.G. Britton , K. Bergmann and E.M. Foster , ‘Cognitive Function and Length of Survival in Elderly Subjects Living at Home’, Australian and New Zealand Journal of Psychiatry, 11 (1977), 113–117: 114.26972010.3109/00048677709159547

155.
On the Registrar General’s index of social classes, see Mike Savage , Social Class in the 21st Century (London: Pelican Books, 2015), 31–35.

156.
Kay *et al.*, *op. cit.* (note 145), 115.

157.

*Ibid*.

158.

*Ibid.*, 115–16.

159.

*Ibid.*, 116.

160.
Office for Population Censuses and Surveys [1970], quoted in Richard Bland , ‘Measuring “Social Class”: A Discussion of the Registrar General’s Classification’, Sociology, 13, 2 (1979), 283–291: 284.

161.

Martin Roth , ‘Senile dementia and its borderlands’, in Jonathan O. Cole and James E. Barrett (eds), Psychopathology in the Aged (New York: Raven Press, 1980), 205–232: 213.

162.

*Ibid*.

163.

*Ibid.*, 214.

164.
Kay *et al.*, *op. cit.* (note 154), 116.

165.

David W.K. Kay , ‘The epidemiology and identification of brain deficit in the elderly’, in Carl Eisdorfer and Robert O. Friedel (eds), Cognitive and Emotional Disturbance in the Elderly (Chicago and London: Year Book Medical Publishers, 1977), 11–27:21.

166.
Garry Blessed, interview with the author (Ponetland, Northumbria, September 2013).

167.
Garry Blessed, interview with Katherine Bick in Katzman and Bick, *op. cit.* (note 152), 82–96: 88.

168.
Bergmann to Davies, *op. cit.* (note 28).

169.
Megan Davies to Stuart Hamilton (20 October 1986), National Archives, FD23/2300.

170.
Stuart Hamilton, ‘Famous Names Test: A Brief Introduction’ (October 1986), National Archives, FD23/2300.

171.

*Ibid.*; see also Marilyn Stevens, ‘Famous Personality Test: A Test for Measuring Remote Memory’, *Bulletin of the British Psychological Society*, 32 (1979), 211.

172.
Anon, *op. cit.* (note 125).

173.
Anon, *op. cit.* (note 131).

174.
Anon, *op. cit.* (note 132).

175.

*Ibid*.

176.

*Ibid*.

177.
D. N. Brookes, C. D. Frith, N. E. Day, R. Levy, M. Swash and C. P. Warlow, ‘Clinical Trials in Alzheimer’s Disease and Related Dementias: A Report Prepared by the Alzheimer’s Disease Clinical Trials Committee’ (1988), National Archives, FD23/2302.

178.

*Ibid*.

179.

Carol Brayne and Paul Calloway , ‘Normal Ageing, Impaired Cognitive Function and Senile Dementia of the Alzheimer Type: A Continuum?’, The Lancet, 331 (1988), 1265–1267:1266.10.1016/s0140-6736(88)92081-82897526

180.

*Ibid*.

181.

Adrienne Little , David Hemsley and Jane Volans , ‘Comparison of Current Levels of Performance and Scores Based on Change as Diagnostic Discriminators Among the Elderly’, British Journal of Clinical Psychology, 26 (1987), 135–140: 136.358064810.1111/j.2044-8260.1987.tb00739.x

182.

*Ibid.*, 138.

183.

*Ibid*.

184.

*Ibid.*, 138.

185.

*Ibid.*, 139–40.

186.

Anon, ‘Senile Dementia of the Alzheimer Type – Normal Ageing or Disease?’, The Lancet, 333(1989), 476–477:476.2563845

187.

*Ibid.*, 476.

188.

*Ibid.*, 477.

189.

Alistair Burns , Brian Lawlor and Sarah Craig , ‘Rating Scales in Old Age Psychiatry’, British Journal of Psychiatry, 180 (2002), 161–167: 161.1182332910.1192/bjp.180.2.161

190.

*Ibid*.

191.

*Ibid*.

192.

*Ibid.*, 163.

193.
Rose, *op. cit.* (note 19), 90.

194.
See Danziger, *op. cit.* (note 19), 179.

195.
Cambrosio *et al.*, *op. cit.* (note 22); Clarke *et al.*, *op. cit.* (note 21).

196.

Martyn Pickersgill , ‘What is Psychiatry? Co-Producing Complexity in Mental Health’, Social Theory and Health, 10, 4 (2012), 328–347:328.2322697510.1057/sth.2012.9PMC3511591

197.
Rose and Abi-Rachid, *op. cit.* (note 25), 137; Timmermans and Berg, *op. cit.* (note 22).

198.
Keating and Cambrosio, *op. cit.* (note 22), 334; Tiago Moriera, Julian C. Hughes, Thomas Kirkwood, Carl May, Ian McKeith and John Bond, ‘What Explains Variations in the Use of Mild Cognitive Impairment (MCI) as a Diagnostic Category?’, *International Psychogeriatrics*, 20, 4 (2008), 697–709.10.1017/S104161020800712618377700

199.
Timmermans and Berg, *op. cit.* (note 22), 22.

200.
Pickersgill, *op. cit.* (note 196), 330.

201.
See Sheila Jasanoff (ed.), *States of Nature: The Co-Production of Science and Social Order* (London and New York: Routledge, 2004).

202.

Raymond Williams , The Long Revolution (Cardigan: Parthian Books: new edition, 2011), 59, 64.

203.
Kay *et al.*, *op. cit.* (note 154), 116.

204.
Savage, *op. cit.* (note 155), 35–7; see also Selina Todd, ‘People Matter’, *History Workshop Journal*, 76, 1 (2013), 259–65; Ross McKibbin, *Classes and Cultures: England 1918–1951* (Oxford: Oxford University Press), 98–105. The focus on class as a problem when it came to identifying cases of Alzheimer’s disease seems to have been a peculiarly British concern. Researchers in the United States also claimed that a poor educational background raised problems, but instead linked it to race and noted that tests such as the MMSE appeared ‘less specific for Blacks than for Whites’. See James C. Anthony, Linda LeReshe, Unaiza Niaz, Michael R. Von Korff and Marshal F. Folstein, ‘Limits of the “Mini-Mental State” as a Screening Test for Dementia and Delirium Among Hospital Patients’, *Psychological Medicine*, 12 (1982), 397–408:402.10.1017/s00332917000467307100362

205.
Rose and Abi-Rachid, *op. cit.* (note 25), 140.

